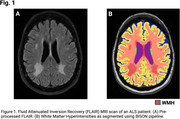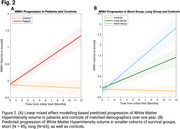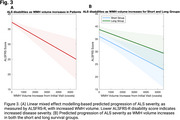# The Utility of White Matter Hyperintensities as A Prognostic Biomarker in Amyotrophic Lateral Sclerosis

**DOI:** 10.1002/alz70856_106677

**Published:** 2026-01-08

**Authors:** Katherine M Chadwick, Isabelle Lajoie, Yashar Zeighami, Sanjay Kalra, Mahsa Dadar, Canadian ALS Neuroimaging Consortium (CALSNIC)

**Affiliations:** ^1^ McGill University, Montréal, QC, Canada; ^2^ Douglas Mental Health University Institute, Montréal, QC, Canada; ^3^ Montreal Neurological Institute, McGill University, Montréal, QC, Canada; ^4^ Neuroscience and Mental Health Institute, Edmonton, AB, Canada; ^5^ University of Alberta, Edmonton, AB, Canada

## Abstract

**Background:**

While sclerosis of the corticospinal and corticobulbar white matter tracts is a key pathological feature of Amyotrophic Lateral Sclerosis (ALS) and previous work in the context of other neurodegenerative diseases has established the link between white matter hyperintensities (WMHs) as magnetic resonance imaging (MRI) markers of white matter damage and disease progression, WMHs remain unexplored in ALS. The present work investigates the relationship between presence and progression of WMHs and disease severity and survival in ALS patients.

**Method:**

We included longitudinal MRI and clinical data of 232 ALS patients and 207 matched controls from the Canadian ALS Neuroimaging Consortium (CALSNIC) (Kalra et al. 2019). T1‐weighted and FLAIR MRIs were used to perform WMH segmentation using BISON pipeline (Figure 1) (Dadar et al. 2021). Patients with survival data (*N* = 110) were categorized as “short” (*N* = 45) or “long” (*N* =  65) survivors based on their time‐to‐outcome from the baseline MRI with a 24‐month cut‐off threshold. Linear mixed effect modeling was employed to investigate the differences in WMH burden and longitudinal progression between the ALS patients as well as survival groups and matched controls, and to assess the relationship between WMH progression and disease severity as measured by ALSFRS‐R. Age and sex were considered as covariates and participant IDs as random effects.

**Result:**

Compared to healthy controls, ALS patients had significantly greater longitudinal WMH progression (911.5 mm3/year, *p* < 0.0001) (Figure 2A). Furthermore, patients in the short survival group experienced greater WMH progression than those in the long survival group (1006 mm3/year, *p* < 0.0001) (Figure 2B). Finally, for every 500 mm3 increase in WMH volume, the ALSFRS‐R scores decreased by a full point (*p* < 0.001) (Figure 3A). While both survival groups experienced increased disease severity with increased WMH volume (*p* < 0.001), their rates of change did not significantly differ (Figure 3B).

**Conclusion:**

This study has shown, for the first time, that ALS patients present with greater WMH progression, and that WMH progression is linked to disease severity and survival in patients, highlighting the utility of WMH as a biomarker of disease progression and prognosis in ALS.